# A fast and accurate enumeration-based algorithm for haplotyping a triploid individual

**DOI:** 10.1186/s13015-018-0129-0

**Published:** 2018-06-01

**Authors:** Jingli Wu, Qian Zhang

**Affiliations:** 1 Guangxi Key Lab of Multi-source Information Mining & Security, Guangxi Normal University, Guilin, 541004 China; 2 College of Computer Science and Information Technology, Guangxi Normal University, Guilin, 541004 China

**Keywords:** Bioinformatics, Sequence analysis, Single nucleotide polymorphism (SNP), Triploid, Haplotype, Minimum error correction with genotype information (MEC/GI), Genotype, Algorithm

## Abstract

**Background:**

Haplotype assembly, reconstructing haplotypes from sequence data, is one of the major computational problems in bioinformatics. Most of the current methodologies for haplotype assembly are designed for diploid individuals. In recent years, genomes having more than two sets of homologous chromosomes have attracted many research groups that are interested in the genomics of disease, phylogenetics, botany and evolution. However, there is still a lack of methods for reconstructing polyploid haplotypes.

**Results:**

In this work, the minimum error correction with genotype information (MEC/GI) model, an important combinatorial model for haplotyping a single individual, is used to study the triploid individual haplotype reconstruction problem. A fast and accurate enumeration-based algorithm enumeration haplotyping triploid with least difference (EHTLD) is proposed for solving the MEC/GI model. The EHTLD algorithm tries to reconstruct the three haplotypes according to the order of single nucleotide polymorphism (SNP) loci along them. When reconstructing a given SNP site, the EHTLD algorithm enumerates three kinds of SNP values in terms of the corresponding site’s genotype value, and chooses the one, which leads to the minimum difference between the reconstructed haplotypes and the sequenced fragments covering that SNP site, to fill the SNP loci being reconstructed.

**Conclusion:**

Extensive experimental comparisons were performed between the EHTLD algorithm and the well known HapCompass and HapTree. Compared with algorithms HapCompass and HapTree, the EHTLD algorithm can reconstruct more accurate haplotypes, which were proven by a number of experiments.

## Background

As a large number of sequencing data are available, the investigation of genetic variations has become one of the main topics in bioinformatics. Single nucleotide polymorphism (SNP), the most widespread form of variation, is believed to be the major genetic cause to phenotypic variability. A sequence of SNPs along a chromosome is referred to as a *haplotype*, which is more important for complete comprehending the complex genetic polymorphisms than isolated SNPs. Increasing evidence shows that haplotypes play a crucial role in studying the variations relating to diseases prediction and gene expression [1]. Therefore, computational methods to infer haplotypes are needed, for determining haplotypes is both time consuming and expensive by direct using biological experiments. In recent decade, the presented computational haplotyping algorithms generally fall into three categories [2]: (1) population haplotyping with genotype data [3, 4]; (2) population haplotyping with fragment data [5]; (3) individual haplotyping with fragment data [6]. In this paper, individual haplotyping problem is studied for a triploid individual.

The problem of individual haplotyping is also called as haplotype assembly problem or haplotype reconstruction problem. It has received extensive study in the recent decade. Most of the existing research results are regarding diploid individuals [1, 7, 8], and there is still a lack of research studies for reconstructing triploid ones. Several algorithms for assembling *K*-individual haplotypes were proposed. Based on the minimum error correction (MEC) model and the minimum error correction with genotype information (MEC/GI) model, Wang et al. [9] and Qian et al. [10] respectively proposed a genetic algorithm and a particle swarm optimization algorithm to reconstruct diploid individual haplotypes, both of which can be adapted to reconstructing the *K*-individual ones. The code length of the two algorithms is very long in practical applications, for it is equal to the number of sequencing SNP fragments. This brings huge solution space to these two algorithms and negatively affects the performance of them. Based on the minimum fragment removal (MFR) model [11], an exact exponential algorithm was introduced by Li et al. [11]. The time complexity of which is $O(2^{2t}m^{2}n+2^{(K+1)t}m^{K+1})$, where *m* denotes the number of SNP fragments, *n* denotes the number of SNP sites, and *t* is the max number of holes covered by a fragment. The algorithm can not perform well with large *m*, *n* and *t*. In 2013, Aguiar et al. [12] introduced the HapCompass model and the minimum weighted edge removal (MWER) optimization for haplotyping polyploid genomes. Algorithm HapCompass aims to remove a minimal weighted set of edges from the compass graph such that a unique phasing may be constructed. The HapCompass algorithm performs on the spanning-tree cycle basis of the compass graph to iteratively delete errors. However, in the same conflict cycle basis, there may be more than one edge having the same absolute value of weight. It may lead to the wrong SNP phasing to select the removed edge randomly. In 2014, Berger et al. [13] described a maximum-likelihood estimation framework HapTree for haplotyping a single polyploid. It can obtain better performance than the HapCompass algorithm [13]. In 2014, based on the MEC model, Wu et al. [14] presented a genetic algorithm GTIHR for reconstructing triploid haplotypes. Since the code length of algorithm GTIHR equals to the number of heterozygous sites in haplotype, the performance of the GTIHR algorithm is negatively affected by haplotype length and heterozygous rate. In this paper, the triploid individual haplotype assembly problem is studied based on the MEC/GI model. An enumeration-based algorithm enumeration haplotyping triploid with least difference (EHTLD) is proposed for solving it. Algorithm EHTLD reconstructs the three haplotypes according to the order of SNP loci along them. For reconstructing the three alleles of a given site, it enumerates three kinds of SNP values by using the site’s genotype, and chooses the kind of value resulting in the minimum difference between the reconstructed haplotypes and the sequenced fragments covering that SNP site. The experimental comparisons were performed between the EHTLD, the HapCompass and the HapTree algorithms. The results proved that the performance of algorithm EHTLD was superior to those of algorithms HapCompass and HapTree. The rest of this paper is arranged as follows. “Definitions and notations” section provides definitions and notations used later. “Algorithm EHTLD” section introduces the EHTLD algorithm. “Experimental results” section presents the experimental results of the EHTLD, the HapCompass and the HapTree algorithms. Some conclusions are drawn in the last section.

## Definitions and notations

**Fig. 1:**
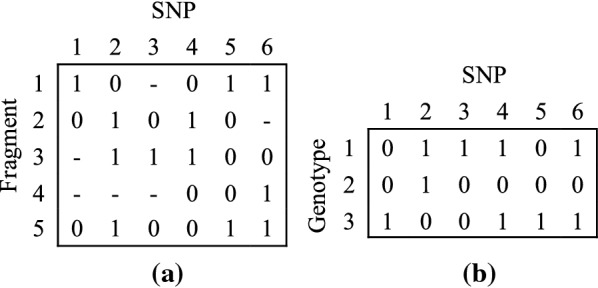
An example of SNP fragment matrix and genotype matrix. **a** SNP fragment matrix *M* , **b** genotype matrix *G*

Triploid somatic cells contain three sets of chromosomes, i.e., a triploid organism has three copies of each chromosome. Since haplotype consists of the sequence of all SNPs along a chromosome, a triploid individual owns three haplotypes. It is commonly regarded that a SNP locus shows merely two possible alleles, hence the major allele can be represented as $\text{`}0\text{'}$ and the minor one can be represented as $\text{`}1\text{'}$. A haplotype can be encoded as a string over a 2-letter alphabet $\{0,1\}$ instead of four real bases {A,T,C,G}. A genotype is the conflation of three haplotypes on the homologous chromosomes. When three alleles at a SNP site have identical values, this SNP site is called a homozygous site, otherwise it is called a heterozygous site. For example, $(000)^{T}$ or $(111)^{T}$ represents the genotype value at a homozygous SNP site, while $(001)^{T}$ or $(011)^{T}$ represents the genotype value at a heterozygous SNP site. Suppose that *m* aligned SNP fragments, coming from three haplotypes of length *n*, are generated by DNA sequencing experiments. Let *M* denote an $m\times n$ SNP matrix over the alphabet $\{0,1,-\}$ (− denotes the value is null). As shown in Fig. 1a, each row represents a SNP fragment, each column represents a SNP site, and each entry *M*[*i*, *j*] denotes the SNP allele of the *i*th fragment at the *j*th SNP site. Let $G=(g_{1},g_{2},\ldots ,g_{n})$ denote the genotype matrix corresponding to *M*, where $g_{j}=(g_{j1},g_{j2},g_{j3})^{T} (g_{jk}\in \{0,1\}, \quad k=1,2,3, \quad j=1,2,\ldots ,n)$ denotes the genotype value at the *j*th SNP site. Figure 1b shows an example of the genotype matrix.

Given a column $M[-,j]\,(j=1,2,\ldots ,n)$ of the matrix *M*, define *r*(*j*) as the set of fragments that cover the *j*th column. Given a row $M[i,-]\,(i=1,2,\ldots ,m)$ of the matrix *M*, let *l*(*i*) indicate the index of the leftmost SNP $j\,(j=1,2,\ldots ,n)$ such that $M[i,j]\ne -$. Given two strings $X=x_{1},x_{2},\ldots ,x_{n}$ and $Y=y_{1},y_{2},\ldots ,y_{n}$, where $x_{j}, y_{j}\in \{0,1,-\}\, (j=1,2,\ldots ,n)$, the distance metric *HD*(*X*, *Y*, *s*, *e*) is defined as Formula (1). Take fragment $f_{1}(10-011)$ and fragment $f_{2}(01010-)$ in Fig. 1a for example. $HD(f_{1}, f_{2}, 2, 5)=3$.

1 $$\begin{aligned} HD(X,Y,s,e)=\sum \limits _{j=s}^e d(x_{j},y_{j}),\quad (1\le s\le e\le n) \end{aligned}$$

where

2 $$\begin{array}{l} d(x_{j},y_{j})= {\left\{ \begin{array}{ll} 1&{} \quad \mathrm{if }~x_{j}\ne -,y_{j}\ne -,\text{ and }\quad x_{j}\ne y_{j}\\ 0&{} \quad \mathrm{otherwise}. \end{array}\right. } \end{array}$$

Let the strings *X* and *Y* be regarded as two SNP fragments, they are said to *compatible* if *HD*(*X*, *Y*, 1, *n*) = 0. The larger *HD*(*X*, *Y*, 1, *n*) is, the greater the probability of fragments *X* and *Y* coming from different chromosome copies or having sequencing errors is. If there are no errors in the data, the rows of *M* can be divided into three classes of *compatible* fragments. Three haplotypes can be reconstructed by assembling the fragments in the three classes. In this situation, the SNP matrix *M* is called *feasible* or *error-free*. Given haplotype $h=(h_{1}, h_{2}, h_{3})\,(h_{k}=(h_{k1}, h_{k2}, \ldots , h_{kn}), k=1,2,3)$ and genotype $G=(g_{1}, g_{2}, \ldots , g_{n}) (g_{j}=(g_{j1}, g_{j2}, g_{j3})^{T}, j=1,2,\ldots ,n)$, if $\sum \nolimits _{k=1}^3 h_{kj}=\sum \nolimits _{k=1}^3 g_{jk} (j=1,2,\ldots ,n), h$ and *G* are regarded as *compatible*.

Based on the above mentioned concepts for the haplotype reconstruction problem, the MEC/GI model can be described as follows [9]:

MEC/GI: Given a SNP matrix *M* and a genotype matrix *G*, correct the minimum number of entries in *M* (0 into 1 and vice versa) so that the resulting matrix is feasible, and the three reconstructed haplotypes are compatible with the genotype *G*.

## Algorithm EHTLD

In this section, the EHTLD algorithm is described. The input consists of a SNP matrix *M* and a genotype matrix *G*. The output is three assembled haplotypes *h* = ($h_{1}$, $h_{2}$, $h_{3}$) of length *n*. In the first step of this algorithm, the matrices *M* and *G* are preprocessed by removing the homozygous SNPs, which do not play a role in assembling haplotypes. Subsequently, enumerates three kinds of values for the *j*th (*j* = 2, 3,…,*n*) SNP site in terms of its genotype, and chooses the one leading to the minimum difference between the reconstructed haplotypes and the fragments covering the *j*th site. After this iteration process is completed, three haplotypes $h'=(h'_{1}, h'_{2}h'_{3}$) having only heterozygous SNP sites are built, for only heterozygous SNPs are remained in the preprocessed matrices. Finally, $h'$ is augmented by inserting the SNPs discarded in preprocessing step and the final haplotypes *h* is obtained. Some key steps of the EHTLD algorithm will be introduced in detail as follows.

### Preprocessing

Since homozygous sites do not contribute to haplotype reconstruction, they are deleted from matrices *M* and *G* to improve the efficiency of assembly. Drop column $j (j=1, 2,\ldots ,n)$ from *G* where $g_{j1}=g_{j2}=g_{j3}$, and the corresponding column is also dropped from matrix *M*. The deleted column *j* ($j=1, 2,\ldots ,n$) is recorded as $g_{j1}$. After dropping columns from matrix *M*, some SNP fragments with only—elements are also deleted, for they are also redundant information. The remained SNPs are all heterozygous sites. For convenience of description, the preprocessed matrices are still denoted by *M* and *G*. Sort the rows of *M* by their *l*(.) values in ascending order. For each column *j* ($j=1, 2,\ldots ,n$) of *M*, calculate set *r*(*j*) which contains the rows covering the *j*th column.

### Enumerating and computing

The EHTLD algorithm iteratively reconstructs each heterozygous site of haplotypes $h'$ = ($h'_{1}$, $h'_{2}$, $h'_{3}$). Each step concerns reconstructing the current empty site, starting from the left first site. Suppose that the first *j* − 1 sites of the three haplotypes $h'$ have already been filled, i.e., ($h'_{k1}$, $h'_{k2}$,…, $h'_{kj-1}$) (*k* = 1, 2, 3, *j* = 2, 3,…, *n*) has been assembled, and the *j*th site is under consideration. The calculating method comprises the following two steps.

1. Enumerating three kinds of possible values according to $g_{j}$:
  - if $\sum \nolimits _{k=1}^3 g_{jk}$ = 1, the three kinds of values are ($h'_{1j}=0, h'_{2j}=0, h'_{3j}=1$), ($h'_{1j}=0, h'_{2j}=1, h'_{3j}=0$) and ($h'_{1j}=1, h'_{2j}=0, h'_{3j}=0$).
  - if $\sum \nolimits _{k=1}^3 g_{jk}$ = 2, the three kinds of values are ($h'_{1j}$ = 0, $h'_{2j}$ = 1, $h'_{3j}$ = 1), ($h'_{1j}$ = 1, $h'_{2j}$ = 0, $h'_{3j}$ = 1) and ($h'_{1j}$ = 1, $h'_{2j}$ = 1, $h'_{3j}$ = 0).
2. Given the *j*th site value ($h'_{1j}$, $h'_{2j}$,$h'_{3j}$), let *D*($h'_{1j}$, $h'_{2j}$, $h'_{3j}$) measure the difference between the reconstructed haplotypes and the fragments covering the *j*th site, as defined in Formula (3). From the three kinds of values enumerated in step (1), choose the one with the minimum *D*(.) value.
  3 $$\begin{aligned} D(h'_{1j},h'_{2j},h'_{3j})=\sum \limits _{i\in r(j)} min\{HD(h'_{k},M[i,-],l(i),j)|k=1,2,3\} \end{aligned}$$

**Fig. 2:**
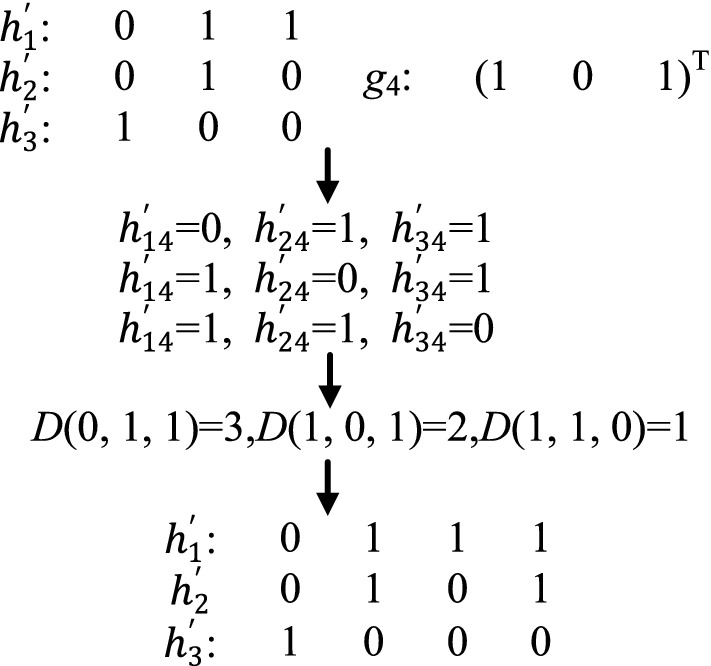
An example for enumerating and computing

In the following, we give an example for enumerating and computing by using the matrices in Fig. 1. As shown in Fig. 2, assume that the first three sites of the three haplotypes $h'$ = ($h'_{1}$, $h'_{2}$, $h'_{3}$) have been reconstructed, i.e., $h'$ = ($h_{1}'$(011), $h_{2}'$(010), $h_{3}'$(100)), and the fourth site is under reconstruction. The genotype of the fourth SNP site is (101)$^{T}$, hence haplotypes $h'$ = ($h'_{1}$, $h'_{2}$, $h'_{3}$) have the following three kinds of possible values on the fourth SNP site: ($h_{14}'$ = 0, $h_{24}'$ = 1, $h_{34}'$ = 1), ($h_{14}'$ = 1, $h_{24}'$ = 0, $h_{34}'$ = 1) and ($h_{14}'$ = 1, $h_{24}'$ = 1, $h_{34}'$ = 0). The values of *D*(0,1,1), *D*(1,0,1) and *D*(1,1,0) are computed respectively according to the fragments in Fig. 1a and the three haplotypes $h'$ = ($h'_{1}$, $h'_{2}$, $h'_{3}$). *D*(0,1,1) = 3, *D*(1,0,1) = 2, *D*(1,1,0) = 1. Because *D*(1,1,0) is the smallest, ($h_{14}'$ = 1, $h_{24}'$ = 1, $h_{34}'$ = 0) is chosen, and $h'$ = ($h_{1}'$(0111), $h_{2}'$(0101), $h_{3}'$(1000)) are reconstructed.

### Augmenting

**Fig. 3:**
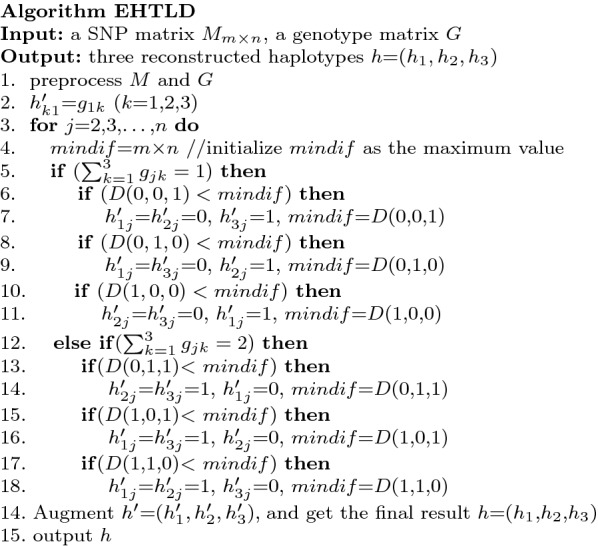
Algorithm EHTLD

The homozygous SNPs that are deleted by preprocessing must be reinserted. The reconstructed haplotypes $h'$ = ($h'_{1}$, $h'_{2}$, $h'_{3}$) are augmented by the bits of the columns removed, and *h* = ($h_{1}$, $h_{2}$, $h_{3}$) are built. For a given position *j*, haplotypes $h'_{1}$, $h'_{2}$ and $h'_{3}$ are inserted with $g_{j1}$ when the discarded column *j* is recorded as $g_{j1}$. Based on the above mentioned steps, the EHTLD algorithm for assembling triploid haplotypes is depicted in Fig. 3.

Now the time complexity of the EHTLD algorithm is discussed. In preprocessing, dropping redundant information and calculating set $r(\cdot )$ take time $O(m\times n)$, sorting the rows of *M* takes time $O(m\times logm)$. During enumerating and computing, three haplotypes with only heterozygous SNP sites are reconstructed, which takes time $O(c \times n\times len)$, here *c* denotes the fragments coverage, and *len* represents the average length of fragments. In augmenting, the discarded columns can be reinserted by scanning the columns only once, which takes time *O*(*n*). In summary, the time complexity of the algorithm is $O(m\times n+m \times logm+ c \times n \times len)$.

## Experimental results

In this section, the EHTLD algorithm is compared with two state-of-the-art algorithms, i.e., the HapCompass [12] and the HapTree [13] algorithms. Algorithms EHTLD and HapCompass were implemented on a Windows 7 and the compiler was Microsoft Visual C# 2012. The Python program HapTree (v0.1), downloaded from http://groups.csail.mit.edu/cb/haptree/, was implemented on a Linux system. All the tests below are conducted on a 64 bit PC with Intel Core i5 2.50GHz CPU and 6GB RAM. One hundred data sets were generated for each parameter setting. The average over 100 runs at each parameter setting was calculated and presented.

The vector error (VE) [13], the reconstruction rate (RR) [1, 9, 15] and the minimum error correction (MEC) score [12] were used to measure the performance of the algorithms. The vector error, generalized from switch error, is a special kind of measurement for evaluating the accuracy of polyploid phasing. Given three reconstructed haplotypes, the vector error is equal to the minimum number of segments on them for which a switch must occur to correspond with the three true haplotypes, i.e., the minimum number of segments a reconstructed phase and the true phase have in common [13].

The reconstruction rate (RR), which measures the similarity degree between the pair of true haplotypes and the pair of reconstructed ones, is a widely adopted index to evaluate diploid phasing [1, 9, 15]. For triploid phasing, we generalized it to calculate the similarity degree between the three true haplotypes and the three reconstructed haplotypes. Assuming that *h* = ($h_{1}$, $h_{2}$, $h_{3}$) are the original haplotypes, and $\hat{h}$ = ($\hat{h}_{1}$, $\hat{h}_{2}$, $\hat{h}_{3}$) are the reconstructed haplotypes. RR is defined as the proportion of nucleotides that are reconstructed correctly, as shown in Formula (4).

4 $$\begin{aligned} RR(h,\hat{h})=1-\frac{min\{\sum \nolimits _{k=1}^3 r_{i_{k}j_{k}}|i_{k},j_{k}\in \{1,2,3\},\prod \nolimits _{k=1}^3 i_{k}=\prod \nolimits _{k=1}^3 j_{k}=6\}}{3n}, \end{aligned}$$

where $$r_{i_{k}j_{k}}$$ = $$HD(h_{i_{k}},\hat{h}_{j_{k}},1,n)$$.

The minimum error correction (MEC) score measures the minimum number of mismatches between the reconstructed haplotypes $\hat{h}$ = ($\hat{h}_{1}$, $\hat{h}_{2}$, $\hat{h}_{3}$) and the SNP matrix *M*, as shown in Formula (5).

5 $$\begin{aligned} MEC(M,\hat{h})=\sum \limits _{i=1}^m\min \{HD(M[i,-],\hat{h}_{k},1,n)|k=1,2,3\}. \end{aligned}$$

To the best of our knowledge, the real triploid haplotype data are not available in the public domain, Aguiar et al. [12] and Berger et al. [13] used computer-generated simulated data. Therefore, simulated data were also used in our experiments. Three simulation haplotypes *h* = ($h_{1}$, $h_{2}$, $h_{3}$) of length *n* were created by using the following method. $h_{1}$ was generated at random firstly. $h_{2}$ was generated by flipping each bit of $h_{1}$ randomly so that the hamming distance between $h_{1}$ and $h_{2}$ was equal to a given parameter *d*. $h_{3}$ also had the same length and $h_{3j}$ was set to $h_{1j}$ or $h_{2j}\,(j=1,2,\ldots ,n)$ with uniform probability. With regard to fragment data, two kinds of sequencing simulators, CELSIM [16] and MetaSim [17], were adopted to generate simulation fragments, and the testing datasets were called as CELSIM instances and MetaSim instances, respectively.

### CELSIM instances

In this section, the evaluation of the EHTLD, the HapCompass and the HapTree algorithms is described by using CELSIM instances. CELSIM was invoked to simulate shotgun sequencing platform. *m*_1_ single SNP fragments and *m*_2_ mate-pair SNP fragments were generated. A single fragment had a length ranging from $f_{min}$ to $f_{max}$, and a mate-pair fragment had a length of *n*/10. The coverage was *c*/2 for both kinds of fragments, and the total coverage was *c*. Reading errors were planted into the fragments with probability $p_{s}$. In practical applications of shotgun sequencing, the values of $f_{min}$ and $f_{max}$ are 3 and 7, respectively, *c* ranges from 5 to 10, and $p_{s}$ ranges between 2 and 5% [2, 18]. In the following tables, algorithms EHTLD, HapCompass and HapTree are abbreviated to EH, HC and HT, respectively.

In Table 1, 12 sets of parameters were set in dealing with error rate $p_{s}$, where *c* = 10, $f_{min}$ = 3, $f_{max}$ = 7, *n* = 100 and *d* = 0.3. It can be seen from this table that algorithm EHTLD can achieve much higher reconstruction rates, smaller vector errors and MEC scores than the HapCompass and the HapTree algorithms in every $p_{s}$ setting. When $p_{s}$ = 0, algorithm EHTLD achieves reconstruction rate of 0.97, which is higher than both HapCompass and HapTree algorithms by about 9.0%, and vector error of 3, which is less than them by 8 times or so. In particular, the MEC score obtained by algorithm EHTLD reaches zero, while those of the other two algorithms are 126 and 57. Although the increase of $p_{s}$ plays stronger negative effect on algorithm EHTLD than on algorithms HapCompass and HapTree, the EHTLD algorithm still obtains better performance than algorithms HapCompass and HapTree with high error rate. When $p_{s}$ = 0.2, the *RR*s of algorithms EHTLD, HapCompass and HapTree are 0.92, 0.89 and 0.88, the vector errors of them are 14, 31 and 26, and the MEC scores of them are 335, 407 and 364, respectively. The three algorithms all execute very efficiently when $p_{s}$ ranges from 0 to 0.2.

**Table 1:** Comparison with different error rates (CELSIM instance)

*p*_*s*_	RR			VE			MEC			Running time (s)		
	EH	HC	HT	EH	HC	HT	EH	HC	HT	EH	HC	HT
0	0.97	0.89	0.89	3	29	27	0	126	57	0.01	0.02	0.01
0.01	0.97	0.89	0.88	3	31	28	17	147	64	0.01	0.03	0.01
0.02	0.97	0.89	0.88	4	30	27	34	152	79	0.01	0.03	0.01
0.03	0.97	0.89	0.90	4	31	26	51	180	83	0.01	0.03	0.01
0.04	0.97	0.90	0.89	4	29	27	69	179	96	0.01	0.03	0.01
0.05	0.97	0.90	0.89	4	29	26	85	194	117	0.01	0.03	0.01
0.06	0.96	0.89	0.88	5	31	26	102	210	132	0.01	0.03	0.01
0.07	0.96	0.89	0.88	5	30	26	122	225	157	0.01	0.03	0.01
0.08	0.96	0.90	0.88	6	29	24	138	238	173	0.01	0.03	0.01
0.09	0.95	0.89	0.87	6	30	28	154	254	181	0.01	0.03	0.01
0.1	0.95	0.90	0.89	7	29	25	172	263	206	0.01	0.03	0.01
0.2	0.92	0.89	0.88	14	31	26	335	407	364	0.01	0.03	0.01

In Table 2, nine sets of parameters were set in dealing with coverage *c*, where *n* = 100, $f_{min}$ = 3, $f_{max}$ = 7, $p_{s}$ = 0.05 and *d* = 0.3. From Table 2 we observe that algorithm EHTLD still obtains the highest reconstruction rate and the smallest vector error and MEC score under different coverage settings. When the coverage is 2, the *RR*s of algorithms EHTLD, HapCompass and HapTree are 0.94, 0.89 and 0.86, the vector errors of them are 10, 30 and 29, and the MEC scores of them are 16, 40 and 19. When the coverage increases, the *RR* of algorithm EHTLD increases gradually, while that of algorithm HapCompass fluctuates between 0.89 and 0.90, and that of algorithm HapTree varies between 0.85 and 0.91. Generally, the increase of coverage plays a positive role in the improvement of algorithm performance, for much more original fragment information can be utilized. However, it is not apparent for algorithms HapCompass and HapTree.

**Table 2:** Comparison with different coverages (CELSIM instance)

*c*	RR			VE			MEC			Running time (s)		
	EH	HC	HT	EH	HC	HT	EH	HC	HT	EH	HC	HT
2	0.94	0.89	0.86	10	30	29	16	40	19	0.01	0.01	0.01
3	0.95	0.89	0.85	9	31	28	25	60	30	0.01	0.02	0.01
4	0.95	0.89	0.91	7	31	28	34	79	38	0.01	0.02	0.01
5	0.96	0.89	0.87	6	30	26	41	96	46	0.01	0.02	0.01
6	0.96	0.89	0.87	5	30	25	52	120	58	0.01	0.02	0.01
7	0.96	0.90	0.89	5	29	26	58	133	65	0.01	0.02	0.01
8	0.96	0.89	0.89	5	30	25	67	157	75	0.01	0.02	0.01
9	0.96	0.89	0.89	4	30	25	75	175	84	0.01	0.03	0.01
10	0.97	0.90	0.89	4	29	26	85	194	117	0.01	0.03	0.01

Table 3 compares the performance of the three algorithms with different haplotype lengths *n*, where *c* = 10, $f_{min}$ = 3, $f_{max}$ = 7, $p_{s}$ = 0.05 and *d* = 0.3. As can be seen from this table, algorithm EHTLD still obtains superior results to the other two algorithms under each parameter setting. With the increase of haplotype length, the three algorithms experience a gradual degradation in the performance. When *n* is 100, the *RR* of algorithm EHTLD is 0.97, which is higher than both HapCompass and HapTree algorithms by about 7.8%, the vector error of algorithm EHTLD is 4, which is less than algorithms HapCompass and HapTree by about 86 and 85%, the MEC score of algorithm EHTLD is 85, which is less than algorithms HapCompass and HapTree by about 56 and 21%, respectively. When *n* is 1000, the *RR*s of them decrease to 0.92, 0.88 and 0.86, the vector errors of them increase to 136, 322 and 314, and the MEC scores of them go up to 479, 1029 and 595, respectively. The running time of the three algorithms increases significantly with the increase of *n*. When *n* = 100, the running time of algorithms EHTLD, HapCompass and HapTree is 0.01, 0.03 and 0.01 s, respectively, while *n* = 1000, it increases to 4.36, 4.67 and 4.51 s, respectively.

**Table 3:** Comparison with different haplotype lengths (CELSIM instance)

*n*	RR			VE			MEC			Running time (s)		
	EH	HC	HT	EH	HC	HT	EH	HC	HT	EH	HC	HT
100	0.97	0.90	0.89	4	29	26	85	194	117	0.01	0.03	0.01
200	0.96	0.89	0.90	12	61	57	136	305	169	0.04	0.16	0.04
300	0.95	0.89	0.88	29	92	90	181	387	230	0.11	0.20	0.09
500	0.93	0.88	0.87	57	156	148	271	573	337	0.56	0.83	0.72
800	0.92	0.88	0.86	100	256	242	398	855	492	2.21	2.59	2.30
1000	0.92	0.88	0.86	136	322	314	479	1029	595	4.36	4.67	4.51

In Table 4, three groups of parameters were set in dealing with single fragment length range $[f_{min}, f_{max}]$, where *c* = 10, $p_{s}$ = 0.05, *n* = 100 and *d* = 0.3. As shown in Table 4, algorithm EHTLD still performs the best under different parameter settings. When $[f_{min}, f_{max}]\,=\,[3,7]$, the *RR*s of algorithms EHTLD, HapCompass and HapTree are 0.97, 0.90 and 0.89, the vector errors of them are 4, 29 and 26, and the MEC scores of them are 85, 194 and 117, respectively. With the decrease of the length of single fragment, the decline of fragments overlap might be disadvantageous for haplotype reconstruction. When $[f_{min}, f_{max}]\,=\,[1,2]$, the *RR*s decrease to 0.94, 0.90 and 0.88, the vector errors increase to 14, 30 and 28, and the MEC scores drop to 65, 86 and 76, respectively. The decrease of the MEC scores explain the shorter the fragments are, the more probability the fragments agree with the reconstructed haplotypes. The change of single fragment length plays little effect on the running time of the three algorithms.

**Table 4:** Comparison with different single fragment length ranges (CELSIM instance)

*f*_*min*_, *f*_*max*_	RR			VE			MEC			Running time (s)		
	EH	HC	HT	EH	HC	HT	EH	HC	HT	EH	HC	HT
[3, 7]	0.97	0.90	0.89	4	29	26	85	194	117	0.01	0.03	0.01
[2, 4]	0.95	0.89	0.88	8	30	28	67	129	90	0.01	0.03	0.01
[1, 2]	0.94	0.90	0.88	14	30	28	65	86	76	0.02	0.03	0.01

Table 5 compares the three algorithms with different hamming distances *d*, where $f_{min}$ = 3, $f_{max}$ = 7, *c* = 10, $p_{s}$ = 0.05 and *n* = 100. It can be seen from this table that the performance of algorithm EHTLD remains relatively stable under different *d*, while that of algorithms HapCompass and HapTree suffers strong negative influence with the increase of hamming distance. For example, when *d* varies from 0.1 to 1.0, the *RR* of the EHTLD algorithm fluctuates between 0.97 and 1.0, while those of the HapCompass and the HapTree algorithms achieve decrease rate up to 35 and 20%, respectively.

**Table 5:** Comparison with different hamming distances (CELSIM instance)

*d*	RR			VE			MEC			Running time (s)		
	EH	HC	HT	EH	HC	HT	EH	HC	HT	EH	HC	HT
0.1	0.99	0.97	0.96	5	9	8	88	109	92	0.01	0.02	0.01
0.2	0.97	0.93	0.94	6	17	16	86	140	106	0.01	0.03	0.01
0.3	0.97	0.90	0.89	4	28	26	85	194	117	0.01	0.03	0.01
0.4	0.97	0.86	0.85	3	38	35	86	260	145	0.02	0.06	0.02
0.5	0.97	0.82	0.84	1	46	42	88	326	201	0.04	0.08	0.03
0.6	0.97	0.79	0.82	1	57	49	89	393	263	0.05	0.10	0.05
0.7	0.98	0.74	0.81	0	71	63	92	478	346	0.07	0.16	0.06
0.8	0.98	0.71	0.80	0	78	72	90	553	421	0.10	0.20	0.08
0.9	0.99	0.67	0.78	0	89	78	90	633	507	0.12	0.25	0.10
1.0	1.00	0.63	0.77	0	94	85	91	722	589	0.15	0.29	0.12

### MetaSim instances

MetaSim was used to simulate 454 sequencing platform. *m* SNP fragments, including ${m_1}\,=\,(1-p_{m})\times m$ single ones and ${m_2}$ = ${p_m} \times m$ mate-pair ones, were generated, where $p_{m}$ denoted the probability of mate-pair fragments and was set to 0.25 in the experiments. A single fragment had an expected length of $f\_len$, and a mate-pair fragment had a length of $3 \times f\_len$. Since each mate-pair fragment consists of two single fragments of the same haplotype, the coverage *c* equals to $[({m_1}+2\times {m_2})\times f\_len]/3\times n$.

Table 6 gives the comparisons with coverage ranging from 5 to 50, where *n* = 100, $f\_len$ = 5, and *d* = 0.3. In Table 7, six sets of experimental results under different haplotype length settings are displayed, where *c* = 20, $f\_len$ = 5, and *d* = 0.3. In Table 8, three instances were generated in dealing with single fragment length $f\_len$, where *n* = 100, *c* = 20, and *d* = 0.3. In Table 9, the test results under different parameter *d* are shown, where *n* = 100, *c* = 20, and $f\_len$ = 5. The experimental results obtained from MetaSim instances indicate that algorithm EHTLD still obtain much higher reconstruction rates, smaller vector errors and MEC scores than the HapCompass and the HapTree algorithms under different $c, n, f\_len$ and *d* settings.

**Table 6:** Comparison with different coverages (MetaSim instance)

*c*	RR			VE			MEC			Running time (s)		
	EH	HC	HT	EH	HC	HT	EH	HC	HT	EH	HC	HT
5	0.94	0.89	0.88	10	30	26	80	134	99	0.01	0.02	0.01
10	0.94	0.90	0.88	8	29	26	144	252	189	0.01	0.02	0.01
15	0.95	0.90	0.88	8	30	26	249	405	287	0.01	0.03	0.01
20	0.95	0.90	0.88	7	30	26	299	513	343	0.02	0.04	0.02
25	0.95	0.89	0.89	7	28	25	383	648	446	0.03	0.05	0.03
30	0.95	0.90	0.89	7	30	25	458	775	531	0.03	0.07	0.03
35	0.95	0.89	0.89	7	30	26	532	903	623	0.05	0.09	0.04
40	0.95	0.90	0.89	7	28	26	600	1028	711	0.07	0.12	0.06
45	0.95	0.90	0.90	7	29	24	700	1193	807	0.10	0.16	0.09
50	0.95	0.90	0.90	7	28	25	758	1293	879	0.13	0.19	0.11

**Table 7:** Comparison with different haplotype lengths (MetaSim instance)

*n*	RR			VE			MEC			Running time (s)		
	EH	HC	HT	EH	HC	HT	EH	HC	HT	EH	HC	HT
100	0.95	0.90	0.88	7	30	26	299	513	343	0.02	0.04	0.02
200	0.93	0.89	0.90	17	62	57	550	985	620	0.24	0.31	0.21
300	0.93	0.89	0.89	25	93	79	788	1441	896	0.56	0.63	0.60
500	0.93	0.88	0.89	45	156	142	1284	2392	1440	2.43	2.83	2.64
800	0.92	0.88	0.89	75	254	238	1998	3791	2254	9.65	10.50	9.89
1000	0.92	0.88	0.88	91	319	305	2484	4731	2804	17.43	17.95	17.47

**Table 8:** Comparison with different single fragment lengths (MetaSim instance)

*f*_*len*	RR			VE			MEC			Running time (s)		
	EH	HC	HT	EH	HC	HT	EH	HC	HT	EH	HC	HT
10	0.96	0.90	0.88	5	30	26	248	404	307	0.01	0.04	0.01
5	0.95	0.90	0.88	7	30	26	299	513	343	0.02	0.04	0.02
3	0.93	0.89	0.89	14	31	28	129	246	201	0.03	0.06	0.03

**Table 9:** Comparison with different hamming distances (MetaSim instance)

*d*	RR			VE			MEC			Running time (s)		
	EH	HC	HT	EH	HC	HT	EH	HC	HT	EH	HC	HT
0.1	0.98	0.97	0.87	4	10	28	347	387	375	0.01	0.01	0.01
0.2	0.96	0.93	0.88	6	18	26	309	427	353	0.01	0.03	0.01
0.3	0.95	0.90	0.88	7	30	26	299	513	343	0.02	0.04	0.02
0.4	0.95	0.86	0.89	6	35	25	294	629	330	0.05	0.11	0.02
0.5	0.94	0.82	0.88	5	48	25	289	752	325	0.09	0.16	0.03
0.6	0.94	0.78	0.88	3	60	26	288	895	320	0.12	0.26	0.03
0.7	0.95	0.75	0.88	3	69	26	296	1060	336	0.18	0.37	0.04
0.8	0.95	0.71	0.88	5	77	26	321	1227	366	0.22	0.42	0.06
0.9	0.96	0.67	0.88	3	86	25	290	1359	333	0.27	0.50	0.09
1.0	0.96	0.63	0.88	2	94	25	277	1523	321	0.35	0.61	0.11

## Conclusion

The minimum error correction with genotype information (MEC/GI) model is one of the important computational models for solving single individual SNP haplotyping problem. In this paper, an enumeration-based algorithm EHTLD is presented for haplotyping a triploid single individual by using this model. Algorithm EHTLD reconstructs the three haplotypes according to the order of SNP loci along them. For a SNP site being reconstructed, the EHTLD algorithm enumerates three possible values in terms of the site’s genotype, and chooses the one leading to the minimum difference between the reconstructed haplotypes and the fragments covering that SNP site. The reconstructed alleles of a SNP site mainly depend on the fragments which cover the site, and are little affected by other former reconstructed alleles. Therefore, the former wrongly reconstructed SNP alleles would not affect the latter reconstructed SNP alleles, i.e., reconstructed errors on the former SNP alleles would not spread to the latter ones. The kind of enumeration strategy can also be apply to reconstruct haplotypes of other ploidy, which will be studied in the future.

Compared with algorithms HapCompass and HapTree by using two kinds of simulated sequencing data, the EHTLD algorithm can get the highest reconstruction rates, the smallest vector errors and MEC scores, which was tested by a number of experiments. In addition, algorithm EHTLD still achieves satisfying performance even with high error rate, low fragment coverage, or long haplotype length. All of these advantages may contribute to the practical application of the EHTLD algorithm when haplotyping a triploid single individual.
